# The Importance of Risk and Subgroup Analysis of Nonparticipants in a Geriatric Intervention Study

**DOI:** 10.1155/2016/2787282

**Published:** 2016-06-30

**Authors:** Elizabeth Rosted, Ingrid Poulsen, Carsten Hendriksen, Lis Wagner

**Affiliations:** ^1^Copenhagen University Hospital, Amager-Hvidovre, 2300 Copenhagen S, Denmark; ^2^RUBRIC, Clinic of Neurorehabilitation, TBI Unit, Rigshospitalet, 2100 Copenhagen Ø, Denmark; ^3^Institute of Public Health, University of Copenhagen and Copenhagen University Hospital, Bispebjerg, 2400 Copenhagen NV, Denmark; ^4^Research Unit of Nursing, Institute of Clinical Research, Faculty of Health Research, University of Southern Denmark, 5230 Odense M, Denmark

## Abstract

*Background*. A major concern in intervention studies is the generalizability of the findings due to refusal of intended participants to actually take part. In studies including ill older people the number of those declining to participate may be large and the concern is therefore relevant.* Objectives*. To compare patients characteristics, rates of acute readmission, and mortality after one and six months among older persons who agreed and those who declined to participate in a randomized controlled trial and to describe subgroups of nonparticipants.* Design*. Comparative study based on a randomized controlled trial.* Setting*. University hospital in the Capital Region of Denmark.* Participants*. Patients ≥70 years discharged home after a short Emergency Department stay. 399 were requested to participate; 271 consented, whereas 128 refused.* Results*. Refusers were more likely to be readmitted (*p* < 0.001) or die (*p* = 0.006). The largest subgroup of refusers described as “too ill” had the highest risk of readmission (OR = 3.00, 95% CI = 1.61–5.47, *p* = 0.001) and of mortality within six months (OR = 3.50, 95% CI = 1.64–7.49, *p* = 0.002). However, this seems not to have affected the results of our randomized study.* Conclusion*. We recommend that intervention studies among older people or other fragile patient groups include analysis of relevant risk and subgroup analyses of refusers.

## 1. Background

The recruitment of participants in intervention studies is challenging and a major concern is the generalizability of the findings due to the intended participants' refusal to participate (“refusers”). In studies including ill older people the group of refusers may be large and the concern is therefore very relevant [1].

The population of older people does not represent a uniform group; on the contrary, they form a diversified group with some being very healthy and fit while others have multi comorbidities and are in need of daily help to manage at home. The generalizability of findings from intervention studies to the wider population of older patients admitted to Emergency Departments (ED) that exclude such participants might therefore be limited. In randomized controlled studies, this is particularly important, as the intervention may have different effects in different subgroups of the study population.

To examine what was reported on older peoples' refusal to participate MEDLINE and CINAHL were searched, containing key words “geriatric assessment” or “older people” and “emergency department” and “refusers” or “non-participants.” Of the studies, we found only few had analyses of differences between refusers and participants or of refuser subgroups as their main focus. The refusal rates were high ranging from 12% to 54% [2–9]. Two studies reported no difference in age [4, 8] and one reported that refusers were two years younger on average [3]. No difference in gender was reported by one study [8], whereas two studies reported a higher proportion of women among refusers than among participants [3, 4]. With regard to health, one study reported that refusers were less likely to be admitted to hospital [3] and another reported that refusers visited the ED more frequently [4].

Concerning risk factors, one study found an increased risk of entering a nursing home among nonparticipants [1], whereas another found that nonparticipants had both a higher mortality rate and a higher rate of nursing home admission [10]. As these studies involved different methods, strategies, age groups, and interventions it is not possible to draw conclusions concerning demographic data or health about older people in the ED refusing to participate in intervention studies.

Refusers' reason for not participating may help to characterize and understand the group; thus two studies concerning disability prevention and preventive home visits found that refusers may be categorized into four groups: “too ill,” “too healthy,” “not interested,” and “other reasons” [1, 10]. Both found that the subgroup of refusers describing themselves as “too ill” had a significantly higher rate of mortality than participants. A qualitative study examining attitudes and views among community-dwelling older people at risk of falling who either rejected referral to or completed a hospital-based falls clinic assessment program found that the two groups expressed opposite opinions regarding most topics; for example, all of the refusers thought that nothing could be done about their falls problem, while all of the accepters thought that something could be done [11].

We found only one intervention study involving older people in EDs that reported reasons for refusal and then only in 25% of the cases. The reasons given were being too ill, being unable to consent, leaving before the completion of tests, and being given medications that could affect patients' mental status during testing [9].

In Denmark 30% of those who use the ED are aged 70 and above [12]. Because of preexisting and complex problems, geriatric assessment and intervention are often required. Though patients may stay for 3 days this is usually not provided and studies have shown that up to 80% of geriatric patients discharged from ED have at least one unrecognized geriatric problem [13, 14]. The proportion of patients declining to participate could be more than one-third of those invited [1, 10]. Thus our aim was to compare baseline data, rates of readmission to hospital, and mortality rates among older persons who accepted and those who declined to participate in a randomized controlled trial and to describe the subgroups of nonparticipants.

## 2. Material and Methods

### 2.1. The Randomized Controlled Trial

The study was designed as a prospective controlled, randomized follow-up study with intervention to evaluate the effect of a short, two-stage nursing assessment and intervention. The intervention was the Standardized Evaluation and Intervention for Seniors At Risk (SEISAR) and comprised a problem-identifying assessment and a problem solving intervention [6]. The assessment consists of a brief, standardized nursing assessment with a checklist of 10 items. Following the assessment, a plan was made to resolve the problems together with the older person. Both the intervention and the control group received visits from the hospitals geriatric nurses where several functional assessment scales were performed.

The trial was registered with Current Controlled Trials Ltd. and the International Standard Randomized Controlled Trial Number (ISRCTN) is ISRCTN08788893. Study design and results have been published elsewhere [15].

### 2.2. Participants

The setting was the ED of a 150-bed University Hospital in the Capital Region of Denmark. The population of the catchment area was 160,000. From February 2009 to February 2011 we included patients aged 70 and above at increased risk of readmission and functional decline and patients who were discharged to their own home within three days. All patients admitted to the ED aged 70 and above were screened using the ISAR screen [16] and considered to be at an increased risk of readmission and functional decline if their score was 2–6. Research nurses in the ED selected patients, however; if patients were not contacted before discharge the nurses phoned them and arranged a visit within three days of discharge. Unlike most intervention studies concerning older people, we did not exclude those suffering from cognitive impairment as they represent approximately one out of four and we hypothesized that they might very well benefit from the intervention. Patients were excluded if they were admitted from a nursing home, were unable to communicate in Danish, were nonresidents of the catchment area, or suffered from terminal cancer.

### 2.3. Enrolment

When high-risk patients (i.e., those with an ISAR score of two or more) were identified and agreed to participate, they were randomly assigned following a simple randomization procedure (computerized random numbers) to either the intervention or control group.

**Figure 1:**
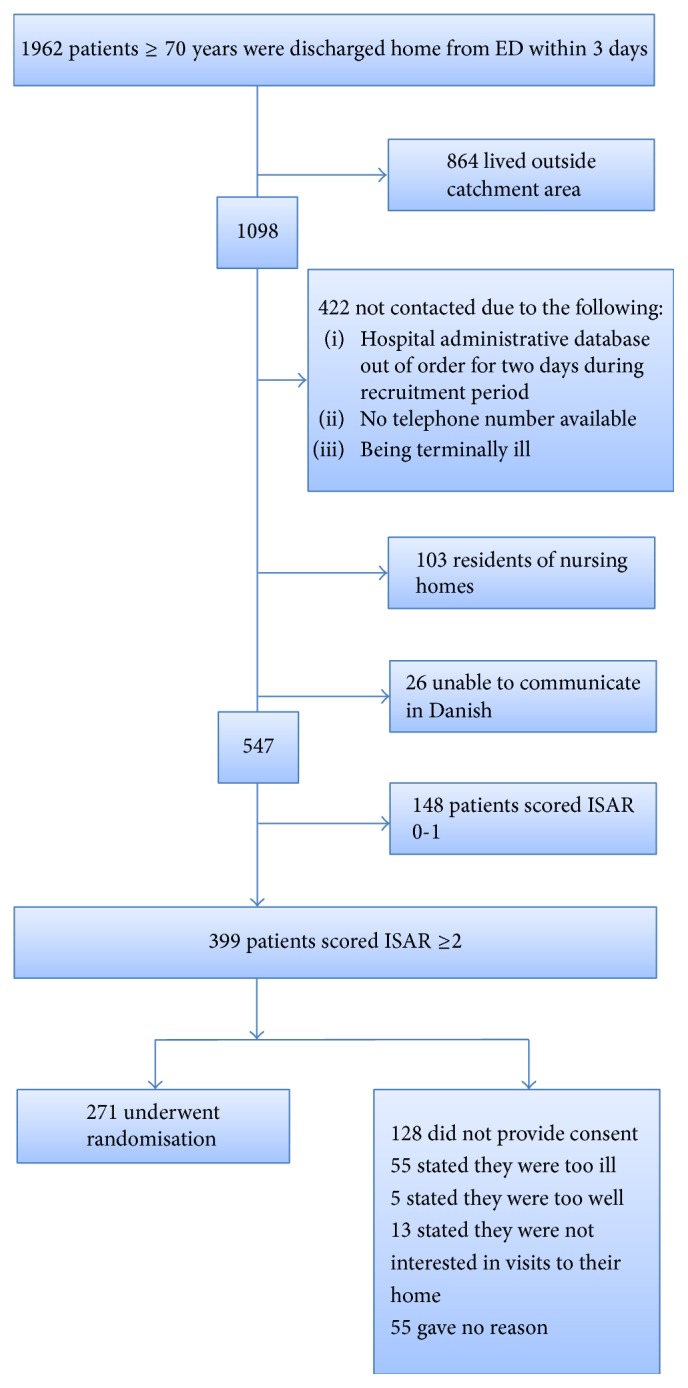
Enrolment of the study population.

The enrolment of the study population is described in detail in Figure 1. If the patient declined to participate the main reasons for refusal were registered and categorized into four groups: “too ill,” “too healthy,” “not interested,” or “gave no reason.”

### 2.4. Measures Outcome

Individuals who were eligible to participate in the study were classified with regard to their status as participants or nonparticipants (i.e., those who actively said no). Sex, age, marital status, medical problems, medication, acute readmission to hospital (admission to hospital not including planned admission such as elective surgery or treatment) within one and six months, and mortality were recorded from the hospital's administrative database.

### 2.5. Analyses

Baseline data was described using mean and observed frequencies. Participants and refusers were compared by chi-square test for categorical variables and by two-sample *t*-test for continuous variables. Whereas more than 20% of the cells had expected counts under five, Fisher's exact test was used. To compare the differences between groups from baseline to follow-up in readmission to hospital and death, logistic regression analyses were used. *p* values under 0.05 were considered significant. All statistical tests were performed using SAS, version 9.0.

### 2.6. Ethics

The ethical principles of the Helsinki Declaration [17] were followed. All participants were given both written and verbal information about the study and they gave written consent if capable of doing so; for confused or demented participants, proxy consents were obtained. No proxy was contacted without the participant's consent. Patients who declined to participate were asked to give consent to their data being used. The study has been approved by the Danish Data Protection Agency (j.nr.2008-41-2768).

This study was supported by TrygFonden and Lundbeck Foundation in Denmark as well as University of Southern Denmark. The funding sources had no role in the study design, data collection, analyses or interpreting data, writing the report, or the decision to submit the paper for publication.

## 3. Results

During the recruitment period, a total of 1,962 patients aged 70 and above were admitted to the ED and discharged within three days. Of these, 547 were screened with ISAR and 399 scored 2 or more and therefore were considered at increased risk of functional decline and readmission (Figure 1). These patients were invited to participate in the study. Two hundred seventy-one patients agreed to participate, whereas 128 refused (32%). The mean age for participants was 81 years and 82 for refusers. No baseline differences were found concerning age, gender, or marital status between participants and refusers (Table 1). The allocation within the ISAR score was equal in both groups and we found no differences in baseline medical problems (Table 1). Significantly more refusers were readmitted to hospital at six months after the initial ED visit (*p* < 0.001). Death rates within periods of both one and six months were significantly higher among refusers (resp., *p* = 0.02 and *p* = 0.006) than of participants (Table 2).

### 3.1. Comparison Involving Subgroups of Refusers

Of all refusers, 43% described themselves as “too ill,” 10% as “not interested, and ” 4% as “too healthy” and 43% gave no reason. In the subgroup analyses, we found more women in the nonparticipating subgroup of “too healthy” and “not interested” than in the participating group (Table 1). There were significantly more married people in the nonparticipating subgroup of “too healthy” than in the participating group. No married people were found in the group “not interested”; we found no significant differences in age. At baseline we found a difference in allocation with patients at risk of readmission and functional decline measured by the ISAR score as among refusers who were “too healthy” or “not interested” no one had a high ISAR score of 4–6 (worst score), while among participants 23% had an ISAR score of 4–6 (Table 1).

During the six-month follow-up the groups who described themselves as “too ill and “not interested” and who “gave no reason” had a significantly higher rate of readmission compared to participants. Moreover, refusers who described themselves as “too ill” were more likely to have been prescribed more than three different medications (*p* = 0.05), they were significantly more at risk of readmission within one (OR = 2.10, 95% CI = 1.07–4.15, *p* = 0.03) and six months (OR = 3.00, 95% CI = 1.61–5.47, *p* = 0.001), and their mortality rate was higher within six months (OR = 3.50, 95% CI = 1.64–7.49, *p* = 0.002).

## 4. Discussion

The focus of this study was to describe the subgroups of nonparticipants and to compare baseline data, as well as rates of readmission to hospital and mortality among those who agreed and those who declined to participate in a randomized controlled trial with follow-up home visits. No baseline differences were found when comparing participants and refusers but we found a significant difference in death rates within periods of both one and six months among refusers (resp., *p* = 0.02 and *p* = 0.006), compared to participants. This indicates that baseline comparisons among participants and refusers are not adequate to indicate if there is a difference between the groups; comparison on follow-up data must also be carried out. We also found that refusers who described themselves as “too ill” and “not interested” and who “gave no reason” had a significantly higher rate of readmission compared to participants and that refusers described as “too ill” were significantly more at risk of readmission within one (OR = 2.10, 95% CI = 1.07–4.15, *p* = 0.03) and six months (OR = 3.00, 95% CI = 1.61–5.47, *p* = 0.001) and their mortality rate was higher within six months (OR = 3.50, 95% CI = 1.64–7.49, *p* = 0.002). This indicates that not only must the baseline comparison be extended with analyses of follow-up data, but also subgroup analyses among different groups of refusers should be performed.

Because there were no differences between the aggregated groups scores for refusers and participants in terms of demographic data, distribution of ISAR score, data concerning medication, or ED visits prior to initial ED admittance at baseline, the finding of differences in readmission and mortality rates was unexpected.

During the six-month follow-up, the refusers experienced a significantly higher rate of readmission to hospital and mortality compared to participants. The study inclusion and exclusion criteria may have influenced the study outcome as well as the distribution of refusers. By not excluding patients with dementia, 41% of our study population were cognitively impaired [15]. ED based interventions in those with dementia have not so far proved effective, as Shaw et al. found no significant difference between intervention and control groups in proportion of those who fell during one year's follow-up [18]; therefore having such a large group of participants with cognitive impairment might have weakened our results. In addition, we excluded those living in a nursing home and as studies imply that they might benefit from multidisciplinary interventions in the ED [19], including those patients may further have influenced our results.

As the difference in readmission and mortality was particularly clear in the subgroup of refusers who described themselves as “too ill” to participate, the finding triggered us to perform a subgroup analysis by reasons for refusing to participate. Other studies found that refusers are disabled at baseline [4, 20, 21] and have a higher risk of mortality [1, 10, 21] which supports our findings. The refusers who described themselves as “too ill” represented 43% of all the refusers in our study and thus weighted more heavily in the analyses of the total group of refusers.

We found no other intervention studies conducted in ED that both included older people and contained analyses of the subgroups of refusers. However, it seems that four subgroups exist, as defined by their reasons for refusal, “too healthy,” “too ill,” and “not interested” and the ones who gave “no reason.” Two randomized trials on disability prevention describe similar subgroups as being “too healthy,” “too ill,” and “not interested,” and giving “no reason” [1, 10]; only the subgroup whose members defined themselves as “too ill” was larger in our study than in these trials (43% versus 12%). This may be explained by our trial including older people who had just been hospitalized and who were at risk of readmission and functional decline, whereas the other studies included older people at home.

The refusers who were “too healthy” were a small group in our study and had a lower risk of readmission or death than the other subgroups. As the targeted group in our intervention study was older people with an ISAR score ≥2, it is only to be expected that the “too healthy” group was relatively small [15]. The largest subgroup of refusers consisted of those who stated they were “too ill” and those giving no reason for refusal. Those declaring themselves to be “too ill” had the highest risk of readmission and death and it seems likely that they had an accurate perception of their own health when describing themselves as “too ill” to participate. The expression “too ill” could also mean that the older people cannot manage extra home visits because they already have professionals coming from the community services assistance during the day. The subgroup that stated they were “not interested” consisted of more women and none of them were married. They had a greater risk of readmission after 6 months compared to the participants and it is well known that living alone itself appears to be associated with a higher risk of falling and constellations of pathologies [22]. The subgroup that gave no reason also had a higher risk of readmission and death compared to the participants. As they gave no reason we do not know why they did not want to participate but, as described by Reuben et al. [23], it may be that the perceived benefit was too low.

A different distribution of refuser subgroups in the studies examining geriatric assessment and intervention may have influenced the results of earlier studies as it seems that overall differences in health status can be hidden if only aggregated analyses of the refusers are carried out. As we do not know if the subgroups in other studies reflect similar dimensions as in ours we may not be able to compare results and this may very well be part of an explanation as to why our results differ.

It is unlikely that the findings of the subgroup analyses would have had implications for the conclusion of our intervention study [15]. Members of the subgroup describing themselves as “too ill” represented 43% of the refusers and they had a higher risk of readmission to hospital or death. As results from our intervention study showed that the participants with an ISAR score of ≥2 had no effect on primary outcomes of the intervention [15], including the refusers may just have strengthened our results.

### 4.1. Limitations

We included participants that stayed between one and three days in the ED and considered this a short stay. We are aware that in some countries a short ED stay is less than 24 hours. In spite of this difference, our results may still be relevant as common to the EDs are that staff qualifications and the treatment are focused on acute care. Another limitation is that 43% of the refusers did not give a reason for refusal. This may be due to the research nurses not being persistent enough when asking for a reason. We do not know whether the subgroup analyses might have given another result if more had answered.

In general, as with all randomized controlled trial subgroup analysis the inference can only be hypothetical and possible pointer to further investigation and future study design. It does not weaken the negative findings of our published study, pointing instead to the possible need for a different intervention with perhaps different subject selection criteria in a future study.

## 5. Conclusion

In conclusion, when only simple aggregated analyses of refusers' baseline data were carried out we noticed no differences either in participants compared to refusers or in the subgroups of refusers.

In order to reveal differences in participants and refusers we thus recommend that intervention studies concerning older people include analyses of follow-up data as well as subgroup analyses of refusers.

## Figures and Tables

**Table 1:** Baseline characteristics of participants, refusers, and subgroups of refusers.

Invited to participate, *n* = 399											
	Participants, *n* (%)			Refusers, *n* (%)							
	Total participants	Total refusers	*p*	Too healthy	*p*	Too ill	*p*	Not interested	*p*	No reason	*p*
	271 (68)	128 (32)		5 (4)		55 (43)		13 (10)		55 (43)	
Age, mean (SD)	82 (6.6)	81 (7.0)		85 (8.7)	0.39	80 (6.5)	0.10	81 (6.4)	0.67	82 (7.4)	0.98
Female	180 (66)	77 (60)	0.22	1 (20)	0.05^*∗*^	33 (60)	0.37	11 (85)	0.23^*∗*^	11 (58)	0.25
Married	61 (23)	26 (24)	0.70	3 (60)	0.08^*∗*^	15 (32)	0.17	0	0.13^*∗*^	8 (18)	0.47
Medical problems					0.59^*∗*^		0.25^*∗*^		0.98^*∗*^		0.23^*∗*^
* Neurological*	18 (7)			1 (20)		2 (4)		0		4 (7)	
* Cardiovascular*	36 (13)			0		7 (13)		1 (8)		8 (15)	
* Respiratory*	18 (7)			0		9 (16)		1 (8)		4 (7)	
* Musculoskeletal*	24 (9)			0		2 (4)		1 (8)		2 (4)	
* Infectious*	32 (12)			1 (20)		6 (11)		2 (15)		13 (24)	
* Other categories *	143 (53)			3 (60)		29 (53)		8 (61)		24 (43)	
ISAR I score			0.91		0.29^*∗*^		0.18		0.06^*∗*^		0.43
* 2*	100 (37)	40 (37)		1 (20)		12 (22)		7 (54)		20 (36)	
* 3*	109 (40)	46 (42)		4 (80)		19 (35)		3 (23)		20 (36)	
* 4*–*6*	62 (23)	23 (21)		0		16 (29)		0		7 (13)	
Medication prescribed > 3	237 (92)	96 (93)	0.66	5 (100)	1.00^*∗*^	42 (100)	0.05^*∗*^	6 (75)	0.15^*∗*^	43 (90)	0.58^*∗*^

^*∗*^Fisher's exact test.

**Table 2:** Comparison of participants, refusers, and subgroups of refusers according to readmission and mortality.

Invited to participate, *n* = 399											
	Participants, *n* (%)			Refusers, *n* (%)							
	Total participants	Total refusers	*p*	Too healthy	*p*	Too ill	*p*	Not interested	*p*	No reason	*p*
	271 (68)	128 (32)		5 (4)		55 (43)		13 (10)		55 (43)	
Readmission to hospital											
* Within 1 month prior to initial ED admission*	40 (15)	17 (13)	0.69	1 (20)	0.56^*∗*^	9 (16)	0.76	2 (15)	1.0^*∗*^	5 (5)	0.25
* Within 1 month after initial ED admission*	41 (15)	29 (23)	0.07	0	1.00^*∗*^	15 (27)	0.04	4 (31)	0.13^*∗*^	10 (18)	0.58
* Within 6 months after initial ED admission*	102 (38)	75 (61)	<0.00	3 (60)	0.37^*∗*^	34 (64)	<0.01	9 (69)	0.02	29 (55)	0.02
Mortality											
* Dead within 1 month*	4 (2)	7 (5)	0.02^*∗*^	0	1.00^*∗*^	3 (5)	0.10^*∗*^	0	1.00^*∗*^	4 (7)	0.03^*∗*^
* Dead within 6 months*	22 (8)	23 (18)	0.01	0	1.00^*∗*^	13 (24)	<0.01	2 (15)	0.30^*∗*^	8 (15)	0.16

^*∗*^Fisher's exact test.
